# The double-domain cytidine deaminase APOBEC3G is a cellular site-specific RNA editing enzyme

**DOI:** 10.1038/srep39100

**Published:** 2016-12-15

**Authors:** Shraddha Sharma, Santosh K. Patnaik, Robert T. Taggart, Bora E. Baysal

**Affiliations:** 1 Departments of Pathology and Thoracic Surgery, Roswell Park Cancer Institute, Elm and Carlton Streets Buffalo, NY, 14263, USA

## Abstract

APOBEC3G is a cytidine deaminase with two homologous domains and restricts retroelements and HIV-1. APOBEC3G deaminates single-stranded DNAs via its C-terminal domain, whereas the N-terminal domain is considered non-catalytic. Although APOBEC3G is known to bind RNAs, APOBEC3G-mediated RNA editing has not been observed. We recently discovered RNA editing by the single-domain enzyme APOBEC3A in innate immune cells. To determine if APOBEC3G is capable of RNA editing, we transiently expressed APOBEC3G in the HEK293T cell line and performed transcriptome-wide RNA sequencing. We show that APOBEC3G causes site-specific C-to-U editing of mRNAs from over 600 genes. The edited cytidines are often flanked by inverted repeats, but are largely distinct from those deaminated by APOBEC3A. We verified protein-recoding RNA editing of selected genes including several that are known to be involved in HIV-1 infectivity. APOBEC3G co-purifies with highly edited mRNA substrates. We find that conserved catalytic residues in both cytidine deaminase domains are required for RNA editing. Our findings demonstrate the novel RNA editing function of APOBEC3G and suggest a role for the N-terminal domain in RNA editing.

The APOBEC3 (A3) family of cytidine deaminases in primates is comprised of seven homologous enzymes that are structurally related to the RNA editing enzyme APOBEC1^1^. A3A, A3C and A3H have a single catalytic domain, whereas A3B, A3D, A3F and A3G have two, N-and C-terminal catalytic domains (NTD and CTD)^2^. Each catalytic domain contains a highly conserved zinc-dependent deaminase motif comprised of HX_1_EX_23_-_28_CX_2_-_4_C (where X is any amino acid)^3^,^4^. The histidine and cysteine residues coordinate the zinc ion whereas the glutamic acid acts as a proton shuttle during the catalytic deamination reaction.

Identification of APOBEC3G (A3G) as a restriction factor for HIV-1 and subsequent studies have revealed that A3 enzymes play an important role in viral restriction^5^,^6^. HIV-1 viral infectivity factor (vif) protein binds A3G and triggers its proteosomal degradation. When the HIV-1 vif protein is absent, A3G is incorporated in HIV-1 particles and inhibits HIV-1 replication in the target cells^6^,^7^. Encapsidation of A3G into HIV-1 particles is essential for its antiviral activity and requires RNA binding by A3G to form a ribonucleoprotein complex with viral proteins^8^,^9^,^10^. Once inside HIV-1 particles, A3G deaminates first-strand HIV-1 cDNA^11^,^12^. Hypermutation of HIV-1 single stranded (ss) DNAs, often within a CC context, plays an important role in the inhibition of HIV-1^13^,^14^, although deamination-independent mechanisms are also involved^15^,^16^. Several models have been proposed for DNA deamination-independent inhibition of HIV-1. These include inhibition of elongation of HIV-1 transcripts by binding to viral genomic RNA^17^, inhibition of ssDNA minus and plus strand synthesis, DNA strand transfer and elongation^15^,^17^. Apart from HIV-1, A3G inhibits LTR-based retroelements by hypermutating their ssDNA and blocking reverse transcription in the cytoplasm^18^. A3G also inhibits SINE (Alu, hY) retroelements by sequestering these RNAs as ribonucleoprotein complexes^19^,^20^.

The mouse genome encodes for a single A3 enzyme (mA3) and it contains two-catalytic domains. *In vitro* studies suggest that mA3 does not induce frequent mutations nor efficiently restrict murine leukemia viruses (MuLV) despite being encapsidated in the viral particles^21^. In contrast, *in vivo* studies with wild-type and mA3-null mice demonstrate that mA3 restricts MuLV. mA3 null mice show increased numbers of infected cells, increased viral loads and reduced latency of MuLV-related T cell lymphomas^22^,^23^. Collectively, these studies suggest that the A3 enzymes may have additional restrictive mechanisms that cannot be explained by the viral ssDNA deamination model of inhibition of retroviruses (reviewed in ref. 6).

A3G has homologous NTD and CTD but only the CTD is active for deamination of ssDNAs^2^,^4^,^24^. Although A3G-CTD catalyzes DNA deamination, antiviral function of A3G requires both domains^24^,^25^,^26^. The zinc-coordinating catalytic residues as well as non-catalytic residues in A3G-NTD are known to bind RNA and this interaction is required for A3G’s binding to the HIV-1 nucleocapsid for recruitment into nascent virions as well as for A3G dimerization. A3G binds to DNA and RNA substrates with similar affinity^27^. Thus far, studies have demonstrated DNA deamination by A3G whereas deamination has not been observed in HIV-1 RNA or synthetic RNA oligonucleotides, thereby, ruling out the RNA editing function of A3G^7^,^11^,^14^,^26^,^27^,^28^.

ssDNA was believed to be the substrate for the A3 family of enzymes^6^,^29^. However, recently we described that APOBEC3A (A3A) induces widespread site-specific C-to-U (C>U) RNA editing of cellular transcripts in pro-inflammatory macrophages and in monocytes exposed to hypoxia and/or interferons^30^. We also showed that the RNA editing function of A3A can be recapitulated by transient overexpression of A3A in 293T cells which causes site-specific RNA editing of thousands of transcripts^31^. Moreover, the majority (75%) of genes that are RNA-edited in the 293T overexpression system are also edited in monocyte-enriched PBMCs (MEPs) exposed to hypoxia and interferon type 1.

To determine if A3G is capable of RNA editing, we transiently overexpressed the protein in 293T cells, a model routinely used by various labs to study A3G function and its mode of HIV-1 restriction^5^,^32^,^33^,^34^,^35^, and then performed transcriptome-wide RNA sequencing (RNA-Seq). Our findings demonstrate a novel RNA editing function of A3G, site-specifically editing hundreds of genes’ mRNAs. Among the validated A3G edited targets are mRNAs of *NMT1, CHMP4B, MAPK1, ACIN1, MED1, NFAT5, RBM14* which are previously known to be involved in HIV-1 replication, assembly, transcription and infectivity. Moreover, our findings suggest that the conserved residues of the zinc dependent deaminase motif of A3G-NTD are required for A3G-mediated RNA editing.

## Results

### RNA-Seq analysis of A3G-expressing cells

To determine whether A3G edits RNAs, we transfected 1 μg of pA3G into 293T cells (293T/A3G, n = 3), confirmed its expression (Supplementary Fig. S1) and performed RNA-Seq, comparing the sequences of 293T/empty vector (control, n = 3) and 293T/A3G transcriptomes. Approximately 19–38 million reads were obtained for each sample in RNA-Seq (Supplementary Table S1). Most of these reads (81–92%) could be mapped by Subread or Tophat alignment softwares. The mapped reads mostly (55–69%) located in coding exons or 3′ or 5′-untranslated regions of genes (Supplementary Table S2). The average depth of coverage by mapped reads among the samples was at least 9 for 28–31 million genomic nucleotide positions. These positions were examined for RNA sequence variation.

Analyses of RNA-Seq data identified single-nucleotide sequence variations in RNA sequences between the two groups of A3G and control transfectants for 712 genomic positions (Supplementary Table S3). At all of these positions, the sequence variation was C>U. Average levels of such putative C>U RNA editing were 0 in all the control samples and >5% in all the A3G transfectant samples for all 712 sites (Supplementary Table S4).

**Figure 1:**
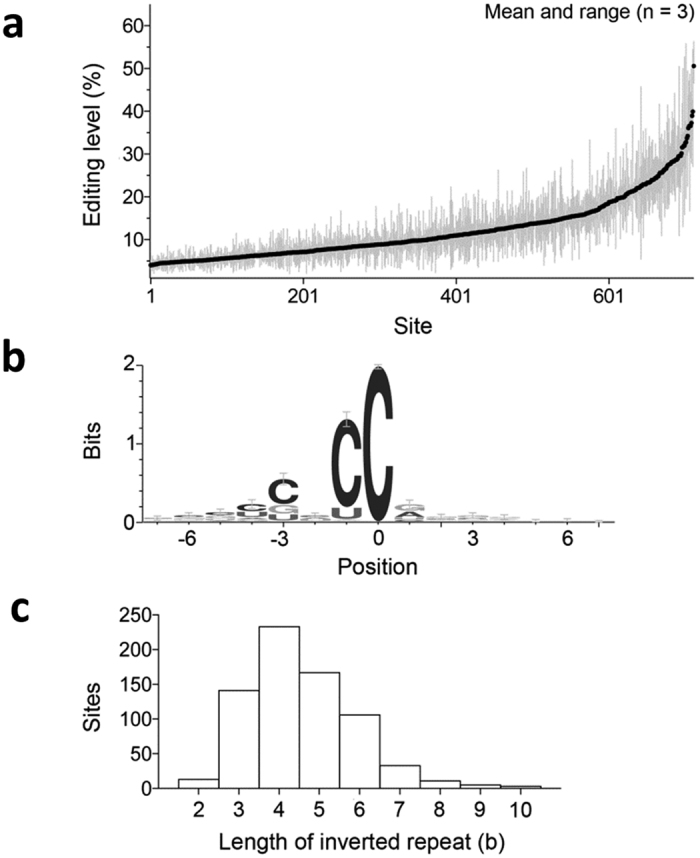
Salient characteristics of C>U RNA editing by A3G in 293T cells. **(a)** Mean and range of editing level at the 712 sites identified as targets for A3G-mediated editing are shown for the three A3G transfectant samples. The sites are ordered by the mean editing level. **(b)** Logo indicating sequence conservation and base frequency for sequences bearing the editing sites (at position 0). **(c)** Histogram of nucleotide lengths (b) of inverted repeat sequences flanking the editing sites.

Average C>U RNA editing levels in the A3G transfectant samples at the 712 sites were between 4% and 51% (mean = 11%, SD = 7%). Editing levels were >20% and >30% for respectively 86 (12%) and 15 (2%) sites (Fig. 1a). 690 (97%) of the 712 sites occur in the known human (RefSeq) transcriptome. Of these sites, 405 (59%) are in known exonic RNA sequences (Table 1). C>U editing of RNA at the 712 sites is predicted to result in 174 (24%) synonymous, 173 (24%) missense and 48 (7%) nonsense changes in RNA translation (Table 1). Protein recoding RNA editing occurred in 221 sites in 217 genes. The 690 editing sites that are in the known transcriptome are transcribed from a total of 635 genes. The highest number of editing sites (4) was seen for two genes, *HCFC1* and *IGF2BP1*. Two and 48 genes respectively had 3 and 2 editing sites (Supplementary Table S4). RNA editing gene targets was enriched for ontologies of methyltransferase activity, nuclear transport, DNA helicase and ubiquitin proteasome pathway (Supplementary Table S5). RNA editing at 90 sites was also catalyzed by A3A (Supplementary Table S6) which causes C>U RNA editing of 4,374 sites in the 293T overexpression system^31^. This finding suggests that although a small overlap exists among the edited sites of A3A and A3G, RNA editing targets of these two enzymes are largely distinct and that A3A has a broader target profile than A3G.

Among the three empty vector control and three A3G overexpressing samples of the study, 18,028 genes were considered as expressed and were analyzed for differential expression. Of the 7,582 (42.1%) genes that were differentially expressed (P < 0.05), 61 and 83 were respectively down- and up-regulated with ≥2 fold-change in the A3G transfectants compared to the controls (Supplementary Table S7). Correlation between editing and gene expression levels was not observed.

To identify common features of sequence contexts of the editing sites, we examined 12 nt sequences flanking the edited C residue. There is a C for 613 (87%), U for 84 (12%), A for 9 or G for 6 sites at the immediate 5′ position of the edited C. This observation and sequence logo analysis (Fig. 1c) suggests [CGU]N[CU]C[AG] (Fig. 1b) as a sequence motif that is commonly targeted by A3G (The residues within brackets are possibilities for a position and the edited C is underlined). CCC, ACC and UCC are respectively seen for 190 (27%), 179 (25%) and 163 (23%) of the 712 editing sites. We previously noticed that Cs edited by A3A are frequently flanked by inverted repeats^30^,^31^. Here, analysis of 25 nts containing the edited C in the middle shows that the edited C is flanked by a pair of inverted repeat sequences of 3–10 nt for 699 (98%) of the sites. Inverted repeat sequences of 4 nt are most common, seen for 233 (32.7%) sites. Inverted repeat sequences of 4 nt or longer flanked over 75% of the A3G-edited Cs, whereas only 28.6% in randomly obtained 25 nt sequences from the human GRCh38 RefSeq transcriptome. GGC, GCC and GGCC are the three most common repeat sequences, seen for 11 (1.5%), 10 (1.4%) and 10 (1.4%) of sites, suggesting that flanking sequence complementarity rather than the sequence per se promotes editing activity. These analyses suggest that, similar to A3A^30^,^31^ both sequence, especially the immediate 5′-nucleotides, and the presence of long flanking inverted repeats play an important role in selection of Cs edited by A3G.

### Validation of A3G-mediated RNA editing

**Figure 2:**
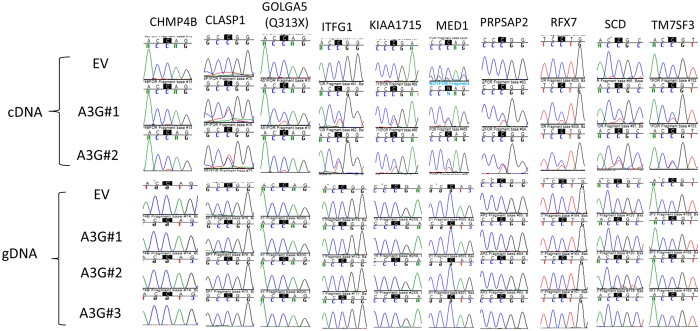
Transient overexpression of A3G induces C>U RNA editing in 293T transfectants. Sanger chromatograms of cDNAs (in duplicate) and genomic DNAs (gDNA) (in triplicate) from control and A3G transfectants. Edited C is shaded black.

To validate novel RNA editing sites identified by the RNA-Seq analysis of 293T/A3G cells, we performed Sanger sequencing of 21 new protein recoding C>U RNA editing sites in 20 genes (Table 2). These genes were selected either because their editing levels were high enough (e.g. *SCD, TM7SF3, CLASP1, PRPSAP2*) to be informative in site-directed mutagenesis studies (below) or they were previously linked to HIV-1 infectivity (*NMT1, CHMP4B, MAPK1, ACIN1, MED1, NFAT5, RBM14*). We validated RNA editing for 21 of 21 sites (100%) by Sanger sequencing in duplicate 293T/A3G transfectants (Fig. 2 and Supplementary Fig. S1). Validation experiments were performed in the same transfectants that were used for RNA seq analysis except for *NMT1, RBM14, MED1* and *MAPK1* which were validated in an independent transfection experiment (Table 2). The validated RNA editing events in HIV-1 related host genes either substantially truncate the protein products due to premature stop codons (*CHMP4B, ACIN1, MED1*) or cause missense changes in evolutionarily conserved amino acids (*NMT1, MAPK1, NFAT5, RBM14*) predicting altered protein function by at least one of three analysis programs (Supplementary Table S8).

### Recombinant human APOBEC3G contains highly-edited mRNAs

**Figure 3:**
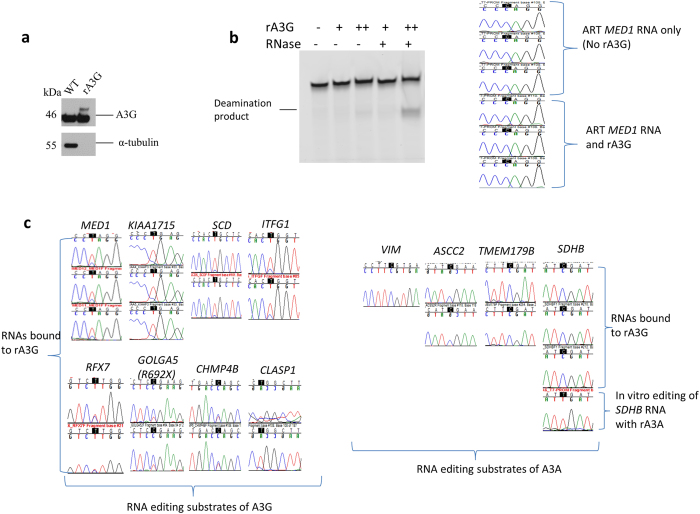
Site-specific C>U RNA editing in mRNAs co-purified with rA3G. **(a)** Immunoblot showing WT A3G expressed in whole cell lysate (20 μg) of 293T cells and recombinant (r) WT A3G (500 ng) obtained from 293T cells (purchased from Origene, Rockville, MD). Full-length blot is presented in Supplementary Fig. S4 **(b)** Cytidine deamination activity of recombinant A3G was examined in an *in vitro* reaction with a 5′ fluorescent dye-labeled ssDNA substrate of 40 nucleotides (left panel). Full-length gel is presented in Supplementary Fig. S4. Sanger chromatograms of cDNAs of *MED1* RNA from *in vitro* RNA editing assay containing rA3G in the presence of 100 nM (+) or 1 μM (++) and ART *MED1* RNA (right panel). (**c**) Sanger chromatograms of cDNAs of A3G substrates (*MED1, KIAA1715, SCD, ITFG1, RFX7, GOLGA5, CHMP4B, CLASP1*) (left panel) and A3A substrates (*VIM, ASCC2, TMEM179B, SDHB)* (right panel) from *in vitro* RNA editing assay containing only rA3G or from *in vitro* RNA editing assay containing *in vitro* transcribed *SDHB* RNA and purified A3A protein (*SDHB* lower panel). Edited cytidines are highlighted in black.

Transient expression of A3G causes editing of RNA suggesting that RNA is a substrate for A3G (Fig. 2). To determine whether A3G can edit RNA *in vitro*, we performed *in vitro* RNA editing assays^30^ with 405 nt RNA of *in vitro* transcribed *KIAA1715* and recombinant A3G. Expression and purification of A3G is difficult in bacterial systems^27^,^36^. Unlike A3A^30^, we were unable to express the full length WT A3G protein in *E. coli*. Therefore, we purchased full length Myc-DDK tagged WT A3G protein from Origene (Rockville, MD) that was expressed in 293T cells and purified via affinity chromatography on anti-DDK column (Fig. 3a). On incubating recombinant A3G with *in vitro* transcribed *KIAA1715* RNA, we observed site-specific RNA editing (Supplementary Fig. S2). A3G is known to co-purify with RNAs^24^,^36^. Therefore, we wanted to confirm the activity of the enzyme and whether the observed RNA editing was in the *in vitro* transcribed RNA or endogenous RNA that might have co-purified with A3G. We performed DNA deamination assay with an artificial 40-oligonucleotide A3G substrate (Fig. 3b, left panel). Recombinant A3G deaminated a 40 nt ssDNA substrate at a higher concentration (1 μM) and when the enzyme was pre-treated with RNase, suggesting that the enzyme is active. To distinguish between editing in the endogenous RNA substrate and *in vitro* generated substrate, we generated an artificial (ART) 216 nt *MED1* RNA substrate that has 2 nt sequence variation (85 nt downstream from the edited C) from the endogenous *MED1* RNA and can be selectively PCR-amplified without co-amplification of endogenous RNA. We incubated purified A3G in the presence or absence (control) of the ART *MED1* RNA. Surprisingly, we detected A3G substrates *KIAA1715, MED1, ITFG1, SCD, RFX7, CHMP4B, GOLGA5* and *CLASP1* as well as A3A substrates *SDHB, VIM, ASCC2* and *TMEM179b* in our control samples that contained recombinant A3G only (Supplementary Fig. S2). We did not detect any of the above RNAs co-purifying with the RNA binding and A>I RNA editing recombinant human ADAR protein that was purchased from Origene and purified using the same method as for A3G (Supplementary Fig. S2). While endogenous A3G substrate RNAs that had co-purified with A3G acquired high levels of site-specific RNA editing, A3A substrate RNAs were not edited at the cytosines catalyzed by A3A (Fig. 3c). However, in an *in vitro* assay with purified A3A, *SDHB* RNA was edited at c.136C>U (Fig. 3c, bottom). We did not observe any editing in the *in vitro* transcribed ART *MED1* RNA either (Fig. 3b). The absence of RNA editing in the artificial transcript may be due to the presence of bound RNAs in the purified protein that may have inhibited RNA deamination. A previous study has shown that in the presence of RNA, DNA deamination is inhibited^25^. Our result, which was obtained by purchasing A3G purified from 293T cells from an independent source, further validates our findings that A3G binds and site-specifically edits its RNA substrates.

### A3G-NTD and CTD are required for A3G’s RNA editing function

**Figure 4:**
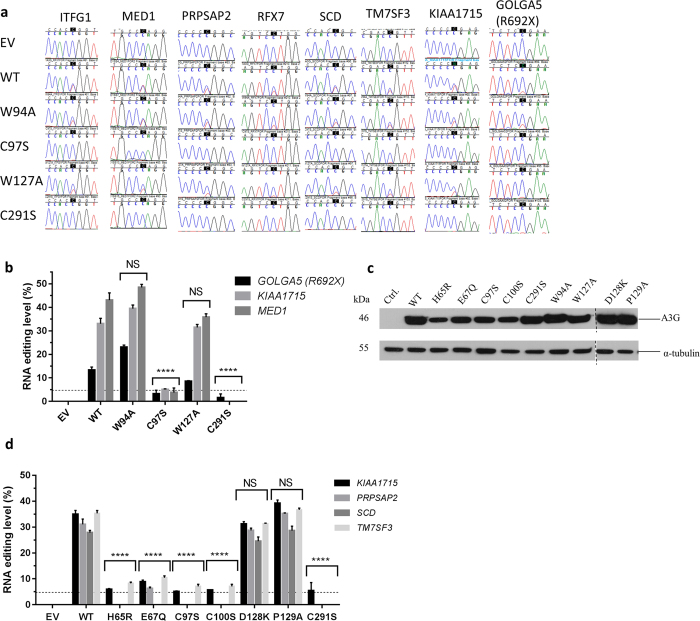
Site-directed mutagenesis of A3G shows requirement for both N- and C–terminal domain zinc dependent deaminase motif residues for site-specific RNA editing. (**a**) Chromatograms of cDNAs (single) from ctrl./A3G transfected 293T cells shows the effect of mutations in A3G-NTD (W94A, C97S, W127A) and A3G-CTD (C291S) on C>U RNA editing (edited C is shaded black) in selected genes. (**b**) Bar graph showing RNA editing level of *GOLGA5, KIAA1715* and *MED1* in A3G-NTD and -CTD mutant transfectants. RNA editing levels (ratio of edited versus total RNA) are calculated by Sequencher^TM^ software. The detection limit for relative height of minor peaks was 4.86% (depicted by a dotted line). Mean and SEM (n = 3) (**c**) Immunoblot showing A3G protein expression in whole cell lysates of 293T cells when transfected with empty vector (control), WT or various A3G-NTD and -CTD mutants. The D128K and P129A mutants were run on separate gels on the same day. The dashed line separates the two gels. Full-length blot is presented in Supplementary Fig. S4 (**d**) Bar graph depicting RNA editing level of *KIAA1715, PRPSAP2, SCD* and *TM7SF3* cDNAs in WT or mutant transfectants. The detection limit is depicted by a dotted line. Mean and SEM (n = 3). Statistical analysis was performed by one-way ANOVA followed by multiple comparisons of RNA editing levels between WT and the mutants. Editing level is considered 0%, when the Sequencher software detects no secondary T peak. ****=adjusted P value ≤ 0.0001; NS = Not Significant.

A3G-NTD binds RNA but is thought to be non-catalytic^26^,^27^. To determine whether one or both domains of A3G are involved in RNA editing, we created substitutions in the conserved zinc-coordinating residues of the CTD (C291S) and NTD (C97S) as well as critical RNA binding residues of the NTD (W94A, W127A)^35^ by site directed mutagenesis. Sanger sequencing of 293T/A3G transfected with the mutant plasmids for eight highly edited genes showed that the most dramatic reduction in RNA editing levels was observed with the C97S and C291S mutants (Fig. 4a and b, Supplementary Fig. S3). C291S completely abolished RNA editing for all genes. C97S completely abolished RNA editing for *SCD, RFX7* and *PRPSAP2*, and was barely detectable for *ITFG1, KIAA1715, MED1* and *TM7SF3* (Fig. 4a and b, Supplementary Fig. S3). These results suggest that both NTD and CTD are required for RNA editing. W127 and to a lesser extent W94 are reported to be essential for RNA interaction, A3G oligomerization and virion encapsidation^34^,^35^. We find that W127A, but not W94A substitutions slightly impaired RNA editing, although the difference was not statistically significant (Fig. 4a and b, Supplementary Fig. S3).

To further examine the role of A3G-NTD in RNA editing, we created additional substitutions in the conserved residues of the zinc-dependent deaminase motif (C100S, H65R, E67Q) and vif-binding non-catalytic residues (D128K and P129A)^37^. On mutating the catalytic residues in A3G-NTD (H65R, E67Q, C97S and C100S), we observed slightly reduced expression of A3G (Fig. 4c), which has been observed previously^26^,^33^,^35^,^37^. As compared to WT A3G, mutations in the A3G-NTD conserved zinc dependent deaminase motif residues abolished RNA editing of *SCD* (Fig. 4d). *PRPSAP2* was minimally edited only by E67Q whereas the editing of *KIAA1715* and *TM7SF3* was markedly reduced (Fig. 4d). Mutations in the non-catalytic residues D128K and P129A did not have a significant effect on RNA editing of different substrates (Fig. 4d). Our results show that the NTD conserved zinc-dependent deaminase motif residues H65, E67, C97and C100 play a role in RNA editing and that both NTD and CTD are required for optimal RNA editing.

## Discussion

In this study, we show that transient expression of A3G in 293T cells causes site-specific C>U RNA editing of hundreds of genes (Supplementary Table S4). Sanger sequencing of 21 selected sites in 20 genes validates RNA editing in all of them (Table 2). Substitutions in the conserved zinc dependent deaminase motif residues in the NTD and CTD of A3G impair or abolish RNA editing (Fig. 4). Recombinant human A3G co-purifies with RNAs that are highly edited at sites identified in our sequencing study (Fig. 3). Our study describes the novel RNA editing function of A3G and suggests the requirement for both catalytic domains for RNA editing. Since RNA editing by APOBEC1, A3A and A3G is site-specific, transcriptome-wide sequencing studies in relevant tissues, cell types or experimental models will be necessary to examine RNA editing abilities of the APOBEC enzymes.

Previous studies of A3G ruled out any catalytic deamination activity of the A3G-NTD^26^,^27^. These studies suggest that the zinc-coordination motif of A3G-NTD is required for RNA binding, encapsidation into nascent viral particles and for A3G dimerization^26^. Moreover, an intact zinc-coordination motif of A3G-NTD is required for inhibiting Alu and HIV-1 retroelements^33^. Mutation in the C97 residue of the NTD cause defects in forming A3G cytoplasmic complexes resulting in decreased HIV-1 virus production^38^. Evolutionary analyses of primate sequences indicate that A3G has undergone a strong positive selection throughout its gene sequence, as evidenced by an excess of non-synonymous over synonymous nucleotide substitutions^39^,^40^. The signature of positive selection on the A3G gene is not limited to the vif-interacting domain but also includes both catalytic active site domains. However, despite this positive selection in the catalytic active site domains, the core catalytic amino acid residues of its NTD that coordinate zinc (e.g. C97, C100 etc) are conserved in the primate A3G genes. The conservation of the catalytic active site in NTD of A3G cannot be readily explained by RNA binding, because both in A3G and in other RNA editing enzymes (APOBEC1 and adenosine deaminases), RNA binding and deaminase domains are distinct^41^. Thus, although previous data suggest an important role for A3G-NTD in restriction of HIV-1, they do not appear to provide an adequate explanation for why the core catalytic residues are conserved in A3G-NTD. Our results show that substitutions in the A3G-NTD catalytic residues (H65, E67, C97, C100) and the CTD C291 residue impair RNA editing. Although A3G-NTD may play a role in the structural integrity of the protein, our results suggest that both catalytic domains are required for RNA editing (Fig. 4) and raise the hypothesis that the NTD catalytic residues may be evolutionarily conserved for RNA deamination.

To determine the requirement for both A3G-NTD and CTD catalytic domains in RNA editing, a structure of the A3G holoenzyme, preferably bound to the ssRNA substrate is imperative. The crystal structure of the full-length A3G or the WT A3G-NTD is not available. However, crystal structures and solution NMR structures of A3G-CTD bound to the ssDNA substrate^42^,^43^,^44^ and the structure of A3G-NTD with 80% sequence similarity to the WT A3G-NTD are available^45^. In the absence of full-length structures of A3G, various models have been proposed for A3G binding to substrate and A3G oligomerization but they are not consistent with each other. In the head-to-head dimer model, the A3G-NTDs interact in an RNA-dependent manner via W127^34^. Since A3G-NTD also binds HIV-1 vif, mutations in D128 and P129 abrogate A3G-vif interaction in this model. In the tail-to-tail model, A3G self-associates via its CTD domain, forming a dimer and then a tetramer to catalyze deamination reactions^25^,^46^. However, subsequent studies showed that oligomerization was not required to retain catalytic activity^47^,^48^. Recently, a head-to-tail dimer structure of A3G has been proposed where ssDNA binds to a continuous groove on the surface and several residues in A3G-NTD including H65, H72, W94, W127, D130 were found to be necessary for ssDNA binding and deamination, whereas W127 was shown to be involved in the interaction between A3G-NTD and CTD^42^. Our results show that mutations in the non-catalytic residues W94, D128 or P129 had no effect whereas mutation in W127 only slightly affected RNA editing. These results suggest that A3G dimerization may not be required for RNA editing and/or ssDNA and RNA binding residues may be different. It is conceivable that zinc dependent deaminase motif residues from both domains of A3G form one active site to catalyze RNA deamination. For example, phospholipase D from Streptomyces sp. and human tyrosyl-DNA phosphodiesterase that belongs to the Phospholipase D superfamily are monomeric enzymes with two similar domains, each containing conserved catalytic residues. In these enzymes, the catalytic residues from both domains form an active site to catalyze hydrolysis of their substrate^49^,^50^.

293T cells that do not express A3G, as well as other overexpression systems have been routinely used by various labs for biochemical/functional studies on A3G and its mechanism of HIV-1 restriction, resulting in hundreds of published articles^5^,^32^,^33^,^34^,^35^. Although overexpression of cytidine deaminase enzymes can lead to spurious RNA editing^51^ or there may be cell-type specific differences in RNA editing, our recent study shows that the majority of the edited sites in 293T cells overexpressing A3A are also edited in monocytes exposed to hypoxia and interferon type 1^31^. Overexpression of A3A or A3G proteins does not lead to random RNA editing, since there is a limited overlap between the editing sites of A3A and A3G in 293T cells, suggesting that these enzymes have distinct RNA substrates. Moreover, we find that A3G prefers to edit transcripts at CC sites, as previously observed with ssDNA templates, and the edited Cs are flanked by 3–10 base long pair of inverted repeats in 98% of the sites (Fig. 1c). These results suggest that like A3A^30^,^31^, A3G prefers RNA substrates with certain sequence/structure characteristics. All sequence verified editing sites are predicted to contain (a) 3 or 4 nt long loop where the edited C is located at the most 3′-end and (b) inverted repeats flanking this putative loop (Table 2). Certain sequences are enriched within the loops (CCC, ACC, UCC) but not in the stem regions. These observations suggest that RNA stem-loop structures may have a role in selecting which cytosines are edited by A3G.

We find that many genes which regulate pathways involved in HIV-1 infection are targets of recoding RNA editing upon A3G overexpression (Supplementary Table S9). We validated A3G-mediated RNA editing of several genes that have been directly linked to HIV-1 infectivity in previous studies. Examples include ACIN1 which is identified as a HIV-1 Tat interacting protein^52^, CHMP4B which is critical for HIV-1 membrane budding via ESCRT pathway^53^, NMT1 which is involved in targeting and assembly of HIV-1 Gag proteins to plasma membrane via N-myristoylation^54^, MAPK1 which interacts with 10 HIV-1 proteins and may play multiple roles in HIV-1 replication^55^. NFAT5 which interacts with an enhancer binding site in LTR of HIV-1 to enable replication in human primary macrophages^56^, MED1, a subunit of mediator complex which plays a critical role in HIV-1 transcription and infectivity^57^,^58^ and RBM14 which encodes a nucleic acid binding protein that associates with XPO1 to export incompletely spliced HIV-1 transcripts^59^. Although combined effects of RNA editing of hundreds of genes on HIV-1 replication is unknown, A3G may alter the host environment by means of RNA editing to antagonize HIV-1 infection. The antagonistic effect of RNA editing may involve reducing the amount or quality of the accessory host proteins that are critical for HIV-1 life cycle or facilitating encapsidation of A3G into nascent virions.

Our study opens up new avenues of inquiry on mechanisms of HIV-1 restriction, A3G-RNA interaction/catalysis and epitranscriptomic regulation by RNA editing. A3G is unusual among the known RNA editing deaminases in that it contains two deaminase domains. Adenosine deaminases acting on pre-mRNAs (ADARs), APOBEC1 and A3A are all single-domain deaminases^41^. Future studies are required to determine the physiological conditions that may induce A3G-mediated RNA editing in primary cells. In conclusion, we demonstrate a novel RNA editing function of A3G and suggest a previously unrecognized function of the A3G-NTD. Identification of A3A previously^30^ and A3G in this study as cellular RNA editing enzymes raises the possibility that other APOBEC3 enzymes may also possess inducible C>U RNA editing functions, which may have implications in immune cell homeostasis and in viral restriction.

## Methods

### RNA sequencing

Directional RNA sequencing libraries were prepared using the TruSeq™ Stranded Total RNA Sample Prep Kit (Illumina^®^, San Diego, CA). Each library, indexed for multiplex sequencing of six libraries per flow lane, was prepared from 1 μg of DNAse I-treated total RNA (Agilent^®^ RIN values of 7.6–9.5) after ribosomal RNA depletion with Ribo-Zero™ Gold reagents. Ten PCR cycles were used during library generation and the modal library fragment size was 300 bp. Paired-end, 101 nt sequencing of libraries was done on HiSeq™ 2000 instrument with TruSeq™ SBS and PE Cluster v3 Kit reagents (Illumina^®^). CASAVA 1.8.2 (Illumina^®^) was used for base-calling and de-multiplexing to obtain raw sequencing reads. Raw read data was deposited in NCBI BioProject with accession number 261741. Reads were filtered and trimmed to remove adapter sequences and poor-quality bases using Trimmomatic 0.33 with options: HEADCROP:12 ILLUMINACLIP: TruSeq3-PE-2.fa:2:30:10:6:TRUE LEADING:5 TRAILING:5 SLIDINGWINDOW:4:15 MINLEN:30. Raw and processed read counts are provided in Supplementary Table S1.

### Mapping of RNA sequencing reads

Reads were uniquely mapped to the GRCh38 human reference genome assembly (Ensembl release 78 of December 2014) using the Subread1.4.5-p1 aligner, with these options specified for the subjunc command: -d 30 -D 400 -uH. Reads were also uniquely mapped with the Tophat 2.0.12 aligner, with these options specified for the tophat2 command: –library-type = fr-firststrand –mate-std-dev = 35 -g 1 -N 3 –no-novel-juncs -r 10 –read-edit-dist 3. Genome indices for these aligners were respectively built with Subread index (options: -BF) and Bowtie2 2.2.3 bowtie2-build commands. Transcriptome index for Tophat was built with tophat2 command using Ensembl’s gene model for the genome assembly. RSeQC 2.4 was used to identify UCSC gene features of genomic regions that the reads mapped to. Mapping statistics are provided in Supplementary Table S2. To count mapped reads at the gene level for the Ensembl gene model, Subread-aligned read data was analyzed with Subread featureCounts with options: -g gene_id -O -p -s 2 -t exon.

### Identification of putative A3G-affected RNA editing sites

The clipOverlap command (option: –poolSize 10000000) of bamUtil master version of 21 September 2014 was used to clip sequence overlaps between read pair-mates in mapped read data. The mpileup command (options: -AB -d 2000 -q 1 -Q 20) of Samtools 0.1.19 was then used to obtain genome-wide read base information from the mapped sequencing data. Custom scripts were used to parse the mpileup output into base calls. Genome positions at which the total A/C/T/G base call was <6 in any of the six samples or <9 in any of the two sample-groups of this study were ignored. Positions were then identified as possibly variant if all samples of at least one sample-group had ≥1 non-reference base calls of the same base-type at a variation level ≥0.02, with group-wide averages of ≥1.3 and ≥0.03 respectively. Variation level was calculated as the ratio of variant base call count to the sum of variant and reference base call counts. Variant positions for which calls for base-types other than the reference and variant constituted ≥1% of all A/C/T/G calls or were >1 in any sample were ignored. Positions with range/variation value of <2 for variation levels across all six samples were also ignored. A two-tailed beta binomial test (IBB 13.06 package for R 3.0.2) comparing variation levels of the two sample-groups was then performed for each of the remaining variant positions. P values obtained with the test were corrected for multiple testing with the Benjamini-Hochberg method. Positions for which the average variation level was >0 for both sample-groups or was <0.04 for ≥1 sample-group, or for which the corrected P value was ≥0.05, or which were not seen with both Subread- and Tophat-aligned data or failed a strand-bias test were ignored. The strand-bias test used base call counts across the group of A3G transfectant samples, and was applied to only those positions for which there were ≥8 calls for each of reference or variant base-types, with <25% of base calls of either base-type from reads of either forward or reverse direction. Failure was deemed if P value in a Fisher exact test that compared reference and variant base calls from reads of the two directions was <0.05. Finally, putative A3G-affected RNA editing sites were identified from the remaining genome positions for which the average editing level for the three A3G trasfectant samples was >0.05. The editing level was the mean of variation levels calculated from Subread- and Tophat-aligned data. The directional nature of the RNA sequencing libraries was used to identify the transcribed chromosomal strand at a genomic position to assign the type of RNA editing. RNA sequences flanking an editing site were deduced from the reference human genome and corrected for homozygous single-nucleotide polymorphisms in the HEK-293T cell-line. The 22 March 2015 release of ANNOVAR tool with its RefSeq-based hg38_refGene database was used to annotate sites with information on gene, gene feature and effect of editing of protein sequence. The list of editing sites with annotations is provided in Supplementary Table S4. The step-wise filtering of genome positions in the process to identify editing sites is detailed in Supplementary Table S3.

### Sanger sequencing to validate RNA editing

PCR amplification and Sequencing primers (Integrated DNA Technologies) are listed in Supplementary Table S10 and purchased from IDT (Integrated DNA Technologies, Inc). PCR reactions were first examined in agarose gel electrophoresis for size verification. PCR products were treated with Exonuclease I and Shrimp alkaline phosphatase (NEB, product numbers M0293S and M0371S, respectively) and then directly used for sequencing on 3130 xL Genetic Analyzer (Life Technologies). Major and minor chromatogram peak heights at a nucleotide position of interest were quantified with Sequencher 5.0/5.1 software (Gene Codes, Ann Arbor, MI), to calculate editing level for the position. As the software identifies a minor peak only if its height is at least 5% of the major peak’s, we consider 0.048 [=5/(100 + 5)] as the detection threshold.

### Gene expression constructs and site-directed mutagenesis

Sequence-verified plasmid constructs in pCMV6 vector for CMV promoter-driven expression of human A3G cDNA, with sequences matching NCBI RefSeq sequences NM_021822.1, for the generation of C-terminal Myc-DDK-tagged A3G proteins were obtained from OriGene (Rockville, MD; product number RC206821). Site-directed mutagenesis of A3G constructs (the nucleotide substitutions and primer sequences are shown in Supplementary Table S10) was performed using Q5 site-directed mutagenesis kit (New England Biolabs, Ipswich, MA). Sequences of cDNA inserts of all of these constructs were verified by Sanger sequencing. Insert-less pcDNA 3.1(+) vector (Life Technologies) plasmid was used for control transfectants.

### Transfection of plasmid DNA

The TLA-HEK293T human embryonic kidney cell line was obtained from Open Biosystems (Huntsville, AL). Thawed 293T cells were passaged at least twice before transfecting the cells at ~50–60% confluency with plasmid DNA using jetPRIME (Polyplus-transfection, New York, NY). Transfection efficiency was 60–80% as assessed by fluorescent microscopy of cells transfected with the pLemiR plasmid DNA (Open Biosystems) for expression of a red fluorescent protein. Cells were harvested 2 days after transfection.

### Immunoblotting of cell lysates

M-PER reagent (Thermo Fisher, Rockford, IL) with 1X Halt protease and phosphatase inhibitor cocktail (Thermo Fisher) was used to prepare whole cell lysates. Reducing and denaturing polyacrylamide gel electrophoresis of 20 μg proteins in Laemmli buffer system was performed on pre-cast, 4–15% gradient polyacrylamide gels (Mini-PROTEAN TGX, Bio-Rad, Hercules, CA). Following electrophoresis, proteins were transferred to polyvinylidene difluoride membrane with a pore-size of 0.2 mm for 7 min at 1.3 A in a Bio-Rad Trans-Blot Turbo apparatus. Membranes were incubated in Tris-buffered 0.15 M NaCl of pH 7.5 with 0.05% v/v TWEEN 20 (TBS-T) (Sigma Aldrich, Saint Louis, MO) and 5% w/v dried, non-fat, cow milk (Carnation, Nestle’, Glendale, CA) for an hour at room temperature. After blocking, the membranes were incubated with primary antibodies overnight at 4 °C. Since A3G is MycDDK tagged at the C-terminus, we used mouse monoclonal anti-DDK^31^ (product number TA50011-100, 4C5, 1:25,000 dilution) to detect A3G. Mouse monoclonal anti-β-actin (product number AM4302, 1:15,000 dilution) or rabbit polyclonal anti-α-Tubulin (product number GTX 110432, 1:10,000 dilution) was used to detect actin or tubulin loading controls, respectively. Following incubation with primary antibodies, the membranes were washed with TBS-T and incubated with Horseradish peroxidase-conjugated, goat anti-mouse or -rabbit IgG antibodies, obtained from Life Technologies at 1:2,000 dilution for one hour at room temperature. The membranes were washed with TBS-T followed by a wash with TBS. Luminata Forte Western HRP Substrate (EMD Millipore) and CL-XPosure auto-radiography films (Thermo Fisher) were used for chemiluminescent detection. Used membranes were stripped using Western blot stripping buffer (catalog number sc-281698) as per the manufacturer’s instructions for re-probing with a different antibody.

### *In vitro* RNA editing assay

*KIAA1715* ORF RNA of 405 nt sequence and *MED1* ORF ART RNA of 216 nt were generated by *in vitro* transcription using cDNA obtained from 293T cells as template and were amplified using KIAA1715-RNA(+T7)-F/KIAA1715-RNA-R primers and MED1-T7-WT-F/MED1-M1-R1 primers, respectively (Supplementary Table S10). The forward primer carried the T7 sequence and MEGAscript T7 Transcription Kit (Life Technologies) was used for *in vitro* transcription as per the manufacturer’s instructions to generate *KIAA1715* and ART *MED1* RNAs. RNA that was isolated from the transcription reaction was treated with DNAse I (Thermo Fisher). The RNAs were purified using RNA clean-up and concentration kit (Norgen Biotek corp., Thorold, ON, catalog # 23600) and its integrity was verified by electrophoresis on an agarose gel. Recombinant human A3G (rA3G) or ADAR containing a C-terminal Myc-DDK tag (product number TP306821, TP319761, respectively) was purchased from OriGene (Rockville, MD). A3G protein expressed in 239T cells was purified on anti-DDK affinity column. *In vitro* assays with rA3G contained 0.84 μM A3G (approximately 2 μg), 50 pg *KIAA1715* RNA/ART *MED1* RNA, 10 mM Tris (pH 8.0), 50 mM KCl and 10 μM ZnCl_2_ (Sigma Aldrich). The reactions were incubated for 2 h at 37 °C. RNA was purified from the reactions using TRIzol (Life Technologies) as per the manufacturer’s instructions. Experiments containing rA3G or ADAR only contained 0.5 μM of the proteins (Supplementary Fig. S2). RT-PCR was performed using the above primers for *KIAA1715* or MED1-M1-R2 primer as the reverse primer for specific amplification of the ART *MED1* RNA and subsequently sequenced using the T7 promoter primer (Supplementary Table S10).

### DNA deamination assay

2 μM of 5′ Alexa Fluor 488 fluorescent dye-labelled ssDNA substrate of 40 bases (5′-ATTATTATTATTATTATTATTCCCAGGATTTATTTATTTA-3′) (Integrated DNA Technologies) was incubated at 37 °C for an hour with 100 nM or 1 μM r A3G (Origene) that was either pre-treated or untreated with Ribonuclease A (Sigma Aldrich) and 2 units of *E. coli* uracil DNA glycosylase (New England Biolabs) in 10 mM Tris (pH 8.0), 50 mM NaCl, 1 mM DTT and 1 mM EDTA in a volume of 10 μl.1 μl of 1 N NaOH was added to the reaction, which was then incubated at 37 °C for 15 min. After adding 1 μl of 1N HCl and 12 μl of 2X loading buffer (80% formamide, 10X TBE), the samples were incubated at 65 °C for 5 min. 10 μl of the reaction was electrophoresed on a 10% Mini-PROTEAN TBE-Urea gels (Bio-Rad). Typhoon 9400 Imager (GE Healthcare) was used to scan the gel in fluorescence mode at 488 nm.

### Statistical analysis

One-way ANOVA test followed by multiple comparisons is used to examine effects of various A3G mutations on RNA editing levels. GraphPad Prism 7.00 was used to perform statistical analysis and draw graphs.

### Other

Sequence logos were created with the WebLogo 3 online tool. Gene set enrichment analyses were performed with PANTHER 9.0 online tool (URL: http://www.pantherdb.org). The edgeR 3.10.2 Bioconductor package was used for differential gene expression analyses. Only those genes with read counts >20 in at least three of the study’s six samples were analyzed; the rowsum.filter and prior.df values for estimateCommonDisp and estimateTagwiseDisp functions were respectively set at 12 and 0.2; and, P values calculated by the Fisher exact test were corrected with the Benjamini-Hocheberg method. Differentially expressed genes with a ≥2-fold change are noted in Supplementary Table S7.

Functional effects of amino acid recoding by RNA editing are predicted by PolyPhen-2 (http://genetics.bwh.harvard.edu/pph2/), Mutation Taster (http://www.mutationtaster.org/) and Mutation Assessor (http://mutationassessor.org/r3/) softwares. Evolutionary conservation of edited amino acid residues is examined using UCSC genome browser (https://genome.ucsc.edu/).

## Additional Information

**How to cite this article:** Sharma, S. *et al*. The double-domain cytidine deaminase APOBEC3G is a cellular site-specific RNA editing enzyme. *Sci. Rep.* **6**, 39100; doi: 10.1038/srep39100 (2016).

**Publisher's note:** Springer Nature remains neutral with regard to jurisdictional claims in published maps and institutional affiliations.

## Supplementary Material

Supplementary Tables

Supplementary Dataset 4

Supplementary Dataset 6

Supplementary Dataset 7

Supplementary Dataset 10

Supplementary Figures

## Figures and Tables

**Table 1:** Gene features and effects on translation codon for A3G-mediated C>U RNA editing sites^a^.

5′ untranslated region			39
Exonic	Synonymous		174
	Non-synonymous	Nonsense	48
		Stop loss	0
		Missense	173
		Unknown	2
	Non-coding RNA		8
3′ untranslated region			227
Intronic	Coding RNA		12
	Non-coding RNA		7
Untranscribed^b^			10
Intergenic			12

^a^As reported by the ANNOVAR annotation tool.

^b^Within 1 kb up- or down-stream respectively of a known transcription start or end site.

**Table 2:** Sanger validation of selected C>U recoding RNA editing sites identified in 293T/A3G cells.

Gene^a^	Chromosomal position^b^	Reference: cDNA and amino acid change^c^	RNA editing level (RNA- Seq)^d^	RNA editing level (Sanger)^e^	Size of putative loop (N.’C)/size of flanking palindrome^f^
*ACIN1*	14: 23063490	NM_001164816:exon6:c.C676T:p.Q226X	0.22	0.23	4/5
*CDC6*	17: 40291480	NM_001254:exon4:c.C472T:p.Q158X	0.08	0.14	3/6
*CHMP4B*	20: 33850995	NM_176812:exon3:c.C412T:p.Q138X	0.07	0.09	4/5
*CLASP1*	2: 121469844	NM_001142273:exon9:c.C829T:p.R277W	0.26	0.45	3 or 4/2
*GOLGA5*^*#1*^	14: 92809464	NM_005113:exon4:c.C937T:p.Q313X	0.09	0.19	4/4
*GOLGA5*^*#2*^	14: 92837408	NM_005113:exon12:c.C2074T:p.R692X	0.10	0.13	3/3
*ITFG1*	16: 47155742	NM_030790:exon18:c.C1816T:p.R606W	0.16	0.38	3/2
*KIAA1715*	2: 175939613	NM_030650:exon10:c.C751T:p.R251X	0.26	0.34	4/5
*MAPK1*	22: 21788373	NM_002745:exon6:c.C740T:p.P247L	0.13	0.11	4/3
*MED1*	17: 39410258	NM_004774:exon17:c.C1963T:p.Q655X	0.27	0.38	4/6
*NFAT5*	16: 69693268	NM_006599:exon12:c.C3389T:p.S1130L	0.12	0.12	4/2
*NFRKB*	11: 129873843	NM_006165:exon20:c.C2527T:p.Q843X	0.07	0.09	7/5
*NMT1*	17: 45061373	NM_021079:exon1:c.C44T:p.P15L	0.23	0.19	4/6(i + 1)
*NVL*	1: 224289696	NM_001243146:exon11:c.C796T:p.Q266X	0.06	0.09	4/4
*PRPSAP2*	17: 18928928	NM_001243936:exon9:c.C802T:p.R268W	0.25	0.35	4/4
*RBM14*	11: 66626504	NM_006328:exon3:c.C1846T:p.R616C	0.22	0.26	4/2
*RFX7*	15: 56096472	NM_022841:exon9:c.C1256T:p.P419L	0.29	0.29	4/3
*SCD*	10: 100352431	NM_005063:exon3:c.C376T:p.R126C	0.24	0.33	4/4
*SGPL1*	10: 70854801	NM_003901:exon5:c.C355T:p.Q119X	0.05	0.11	4/4(i + 1)
*SUCLA2*	13: 47973266	NM_003850:exon5:c.C661T:p.Q221X	0.13	0.17	4/6
*TM7SF3*	12: 26974149	NM_016551:exon12:c.C1529T:p.P510L	0.30	0.39	4/2

^a^Genes selected from bioinformatic analysis for verification.

^b^Based on the UCSC hg19 genome assembly.

^c^NCBI reference sequences and the editing related changes at cDNA and protein levels.

^d^Editing levels in RNA-Seq are averages estimated by Tophat and Subread alignment softwares.

^e^Editing levels in 293T/A3G cells are calculated from Sanger traces via Sequencher^TM^ software (2 or 3 replicates).

^f^Indicates size of putative loop where the edited C is at the most 3′-end of it, and the size of immediately flanking inverted repeats (i + 1 indicates interruption in complementarity by 1 unpaired nucleotide).
