# Supplementary material for: The double-domain cytidine deaminase APOBEC3G is a cellular site-specific RNA editing enzyme
Source: Sci Rep. 2016 Dec 15;6:39100. doi: 10.1038/srep39100 (PMC5156925; doi:10.1038/srep39100)
Supplement: Supplementary Tables [file srep39100-s1.pdf]

The double-domain cytidine deaminase APOBEC3G is a cellular site-specific RNA editing enzyme

Shraddha Sharma[1], Santosh K. Patnaik[2], Robert T. Taggart[1], Bora E. Baysal[1*]

Departments of [1]Pathology and [2]Thoracic Surgery, Roswell Park Cancer Institute

Buffalo, NY, 14263

Supplementary Table S1
Read pairs in raw and processed RNA sequencing data

Transfectant	*Replicate*[a]	*Raw data*	*Processed data*[b]
Control	1	30953907	29429364
Control	2	36784988	35272514
Control	3	25910693	24941552
APOBEC3G	1	19218020	18505037
APOBEC3G	2	38280664	37011159
APOBEC3G	3	26523549	25427410

[a]RNA from three each of replicate empty vector and APOBEC3G expression plasmid transfectants was sequenced

[b]Sequencing data after filtering/trimming of reads with Trimmomatic tool

Supplementary Table S2
Mapping of RNA sequencing data

			Feature of region that reads mapped to (%)[b]			
Transfectant	*Replicate*[a]	*Reads mapped (%)*	*CDS exon*	*5' UTR exon*	*3' UTR exon*	*Intron*
		Subread aligner				
Control	1	84	43	5	16	33
Control	2	81	37	4	14	40
Control	3	81	41	5	16	35
APOBEC3G	1	85	46	4	17	30
APOBEC3G	2	91	45	4	18	29
APOBEC3G	3	90	47	4	18	27
		Tophat aligner				
Control	1	89	44	8	16	30
Control	2	91	37	8	14	37
Control	3	92	40	9	15	32
APOBEC3G	1	88	47	6	16	27
APOBEC3G	2	91	48	5	18	26
APOBEC3G	3	90	49	6	17	25

[a]RNA from three each of replicate empty vector and APOBEC3G expression plasmid transfectants was sequenced

[b]RefSeq gene features. *CDS* coding sequence; *UTR* untranslated region

Supplementary Table S3
Number of genomic nucleotide positions along different steps of analysis of aligned RNA sequencing reads for identification of RNA editing sites[a]

Exclusion condition in order of application	*Subread-aligned data (1392515508 positions[a])*		*Tophat-aligned data (1295241947 positions)*	
	Positions excluded	*Remaining positions*	*Positions excluded*	*Remaining positions*
1. ≥6 total A/C/G/T base calls in every sample	1363314048	29201460	1262410710	32831237
2. ≥9 total A/C/G/T base calls in every sample-group	1411821	27789639	1630599	31200638
3. No reference base call in any sample	13433	27776206	8728	31191910
4. No variant base call in any sample	26482152	1294054	27566236	3625674
5. ≥1 variant base calls in each sample of any sample-group with average ≥1.3	1273317	20737	3567418	58256
6. ≥2% variation level in all samples of any sample-group with average ≥3%	0	20737	0	58256
7. <99% of total A/C/G/T base calls for reference and variant bases or >1 call for remaining bases in any sample	351	20386	12059	46197
8. Among all samples minimum variation level >0% or maximum variation level >99%	17433	2953	32542	13655
9. Among all samples range:average variation level <2	754	2199	4046	9609
10. Average variation level >0% in both sample-groups	1015	1184	6444	3165
11. Average variation level >4% in neither sample-group	95	1089	373	2792
12. Adjusted P ≥0.05	84	1005	1250	1542
13. Absent in similarly filtered data for the other aligner	284	721	821	721
14. Fails strand-bias filter	0	721	0	721
15. Mean of average APOBEC3G sample-group variation level calculated with Subread- and Tophat-aligned data is ≤5%	9	712	9	712

[a]Positions with ≥1 base call in ≥1 sample

Supplementary Table S5
Enrichment for ontologies of genes for which APOBEC3G-mediated C>U RNA editing was identified[a]

Ontology term	All genes[b]	Affected genes: Observed	Affected genes: Expected	Enrichment	P^c
Panther GO-Slim molecular function					
methyltransferase activity (GO:0008168)	129	15	4.06	3.70	3.65E-03
helicase activity (GO:0004386)	127	13	4.00	3.25	4.33E-02
ubiquitin-protein ligase activity (GO:0004842)	161	15	5.07	2.96	4.08E-02
ligase activity (GO:0016874)	415	32	13.06	2.45	8.71E-04
receptor activity (GO:0004872)	1636	19	51.48	0.37	1.32E-05
receptor binding (GO:0005102)	980	10	30.84	0.32	1.50E-03
peptidase activity (GO:0008233)	630	5	19.83	0.25	1.17E-02
Panther GO-Slim biological process					
nuclear transport (GO:0051169)	85	12	2.67	4.49	5.03E-03
DNA repair (GO:0006281)	172	18	5.41	3.33	3.03E-03
chromatin organization (GO:0006325)	250	24	7.87	3.05	5.14E-04
organelle organization (GO:0006996)	571	38	17.97	2.11	4.16E-03
immune system process (GO:0002376)	1391	22	43.77	0.50	3.18E-02
Panther GO-Slim cellular component					
extracellular region (GO:0005576)	662	3	20.83	<0.2	5.73E-05
Panther protein class					
DNA helicase (PC00011)	55	10	1.73	>5	2.70E-03
methyltransferase (PC00155)	124	15	3.90	3.84	2.79E-03
helicase (PC00115)	126	13	3.97	3.28	4.83E-02
ligase (PC00142)	386	27	12.15	2.22	2.77E-02
receptor (PC00197)	1596	23	50.22	0.46	1.54E-03
signaling molecule (PC00207)	1083	13	34.08	0.38	4.73E-03
cell adhesion molecule (PC00069)	507	3	15.95	<0.2	1.72E-02
Panther pathways					
Ubiquitin proteasome pathway (P00060)	63	11	1.98	>5	1.12E-03

[a]Analysis requiring ≥2-fold enrichment using all 655 genes whose identifiers were accepted by the PANTHER tool

[b]Number of all genes with annotated with the ontology term in the in PANTHER annotation database

[c]Adjusted for multiple testing with the Bonferroni method

Supplementary Table S8

Predicted effects of protein recoding by A3G-mediated RNA editing in selected HIV-1 related host genes

Gene	cDNA	Protein	PolyPhen-2	MutationTaster	Mutationassessor functional impact	Evolutionary conservation*
ACIN1	C676T	Q226X	N/A	disease causing	N/A	N/A
CHMP4B	C412T	Q138X	N/A	disease causing	N/A	N/A
MAPK1	C740T	P247L	Probably damaging	disease causing	medium (2.015)	Yes (Fish)
MED1	C1963T	Q655X	N/A	disease causing	N/A	N/A
NFAT5	C3389T	S1130L	Probably damaging	disease causing	Medium (2.125)	Yes (Sarcopterygii)
NMT1	C44T	P15L	Benign	disease causing	low (1.04)	Yes (Mammal)
RBM14	C1846T	R616C	Probably damaging	disease causing	low (0.805)	Yes (Sarcopterygii)

*Based on Comparative Genomics/Conservation analysis in UCSC genome browser. The phylogenetically most distant group of species that shows conservation is in parenthesis. N/A= not analyzed/analyzable for nonsense codons

Supplementary Table S9

Genes that acquire recoding C>U RNA editing regulate cellular pathways involved in HIV-1 infection.

ESCRT pathway, HIV RNA trafficking [1]	RNA, DNA replication, modification [2]	NF-KB Pathway [3]	Chromatin modifiers [4]	Interacts with HIV RNA/ Proteins [5]	Nuclear membrane/ pore [6]	Cytoskeleton [7]	Mitochondria [8]	Proteasome [9]	MAPK signaling [10]	Golgi [11]
ZNF142	MED1, MED15, MED28	USP34	PAXIP1	ACIN1	LBR	PDLIM3	DNM1L	PSMD4<br>PSMC3	LAMTOR3	GOLGA3, GOLGA5
CHMP4B	PAPOLG	TAB1	NFRKB	WDR48	NUP205	CLASP1	SUCLA2		MAPK1	
MID2<br>RBM14	CDC6<br>SETD2	UBE2L3<br>UBE2N (UBC13)	CTCF<br>SMARCA4	NSUN5<br>NMT1	NUP54	CAMSAP1				
VPS37A	NFAT5		KMT2A, KMT2C, KMT2D	HSPA5						
ATRN<br>AP2M1	NEIL3<br>HUWE1		BAZ1B<br>CHD7, CHD8							
SIN3A<br>SMG6	EIF3I		CBX6<br>ARID4A<br>PHF2<br>ATF7IP<br>JADE1<br>ASH1L<br>EP300							

Supplementary references

1. Votteler, J. & Sundquist, W. I. Virus budding and the ESCRT pathway. *Cell host & microbe* **14**, 232-241 (2013).

2. Ruiz, A. *et al*. Characterization of the influence of mediator complex in HIV-1 transcription. *J. Biol. Chem.* **289**, 27665-27676 (2014).

3. Griffin, G. E., Leung, K., Folks, T. M., Kunkel, S. & Nabel, G. J. Activation of HIV gene expression during monocyte differentiation by induction of NF-kB. (1989).

4. Lusic, M. & Giacca, M. Regulation of HIV-1 latency by chromatin structure and nuclear architecture. *J. Mol. Biol.* **427**, 688-694 (2015).

5. Mukhopadhyay, A., Maulik, U. & Bandyopadhyay, S. A novel biclustering approach to association rule mining for predicting HIV-1–human protein interactions. *PLoS One* **7**, e32289 (2012).

6. Di Nunzio, F. *et al*. Human nucleoporins promote HIV-1 docking at the nuclear pore, nuclear import and integration. *PLoS One* **7**, e46037 (2012).

7. Bukrinskaya, A., Brichacek, B., Mann, A. & Stevenson, M. Establishment of a functional human immunodeficiency virus type 1 (HIV-1) reverse transcription complex involves the cytoskeleton. *J. Exp. Med.* **188**, 2113-2125 (1998).

8. Groppelli, E., Starling, S. & Jolly, C. Contact-induced mitochondrial polarization supports HIV-1 virological synapse formation. *J. Virol.* **89**, 14-24 (2015).

9. Sheehy, A. M., Gaddis, N. C. & Malim, M. H. The antiretroviral enzyme APOBEC3G is degraded by the proteasome in response to HIV-1 Vif. *Nat. Med.* **9**, 1404-1407 (2003).

10. Jacque, J. M. *et al*. Modulation of HIV-1 infectivity by MAPK, a virion-associated kinase. *EMBO J.* **17**, 2607-2618 (1998).

11. Liu, S., Dominska-Ngowe, M. & Dykxhoorn, D. M. Target silencing of components of the conserved oligomeric Golgi complex impairs HIV-1 replication. *Virus Res.* **192**, 92-102 (2014).
