# Supplementary material for: The double-domain cytidine deaminase APOBEC3G is a cellular site-specific RNA editing enzyme
Source: Sci Rep. 2016 Dec 15;6:39100. doi: 10.1038/srep39100 (PMC5156925; doi:10.1038/srep39100)
Supplement: Supplementary Figures [file srep39100-s6.pdf]

The double-domain cytidine deaminase APOBEC3G is a cellular site-specific RNA editing enzyme

Shraddha Sharma[1], Santosh K. Patnaik[2], Robert T. Taggart[1], Bora E. Baysal[1*]

Departments of [1]Pathology and [2]Thoracic Surgery, Roswell Park Cancer Institute Buffalo, NY, 14263

Supplementary Figure 1

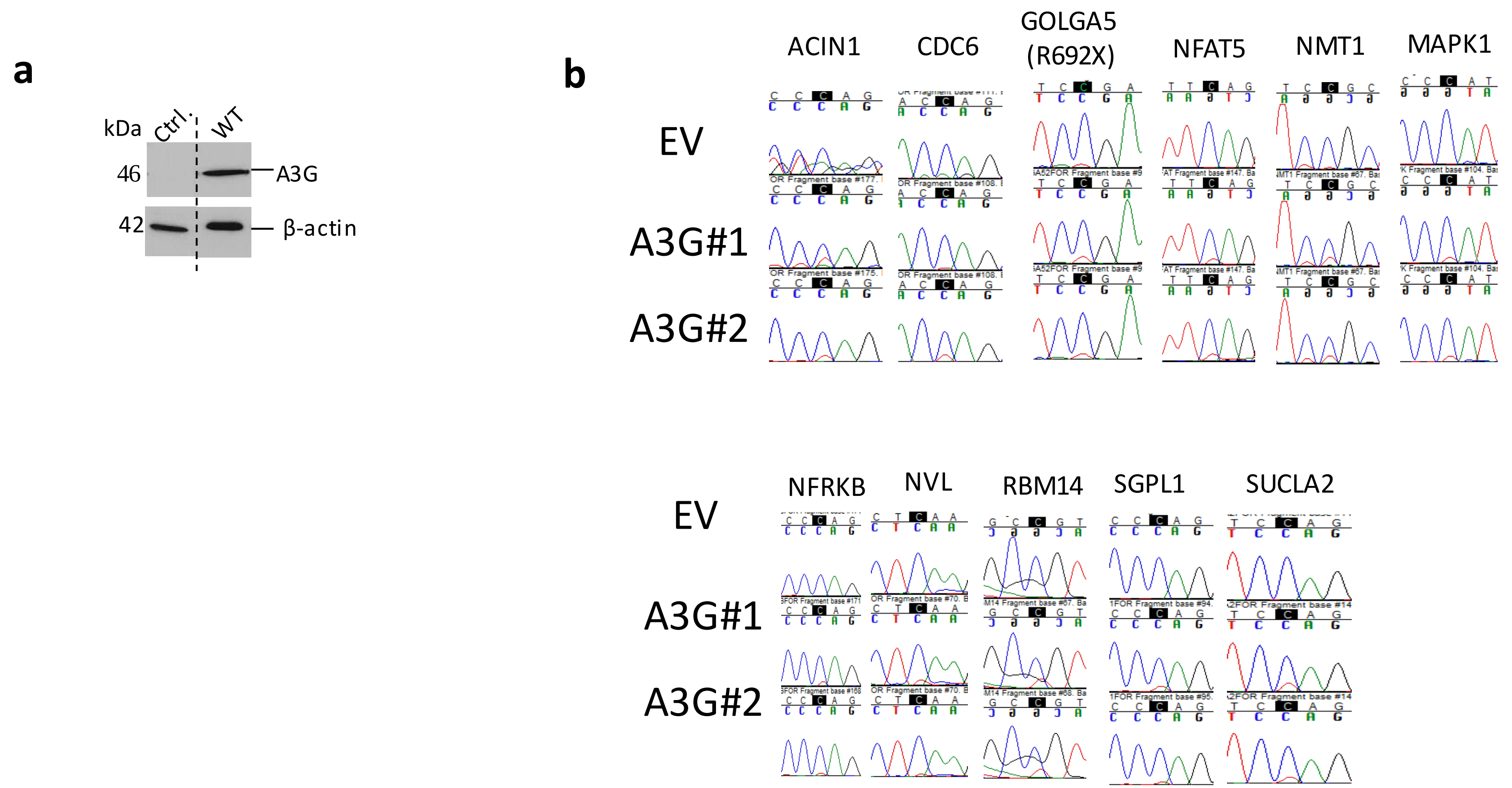

Supplementary Fig. 1: Transient overexpression of APOBEC3G induces C>U RNA editing in 293T transfectants. (a) Immunoblot showing the expression of WT A3G in empty vector (ctrl.) and A3G in whole cell lysates (20 µg) of transfectants. β-actin is a loading control. Both lanes are part of the same gel, but other lanes have been cropped out and the dashed line represents the cropped region. **(b)** Chromatograms of cDNA (in duplicate) RT-PCR products from control and A3G transfectants confirms site-specific C>U RNA editing (edited C is shaded black) in selected genes.

Supplementary Figure 2

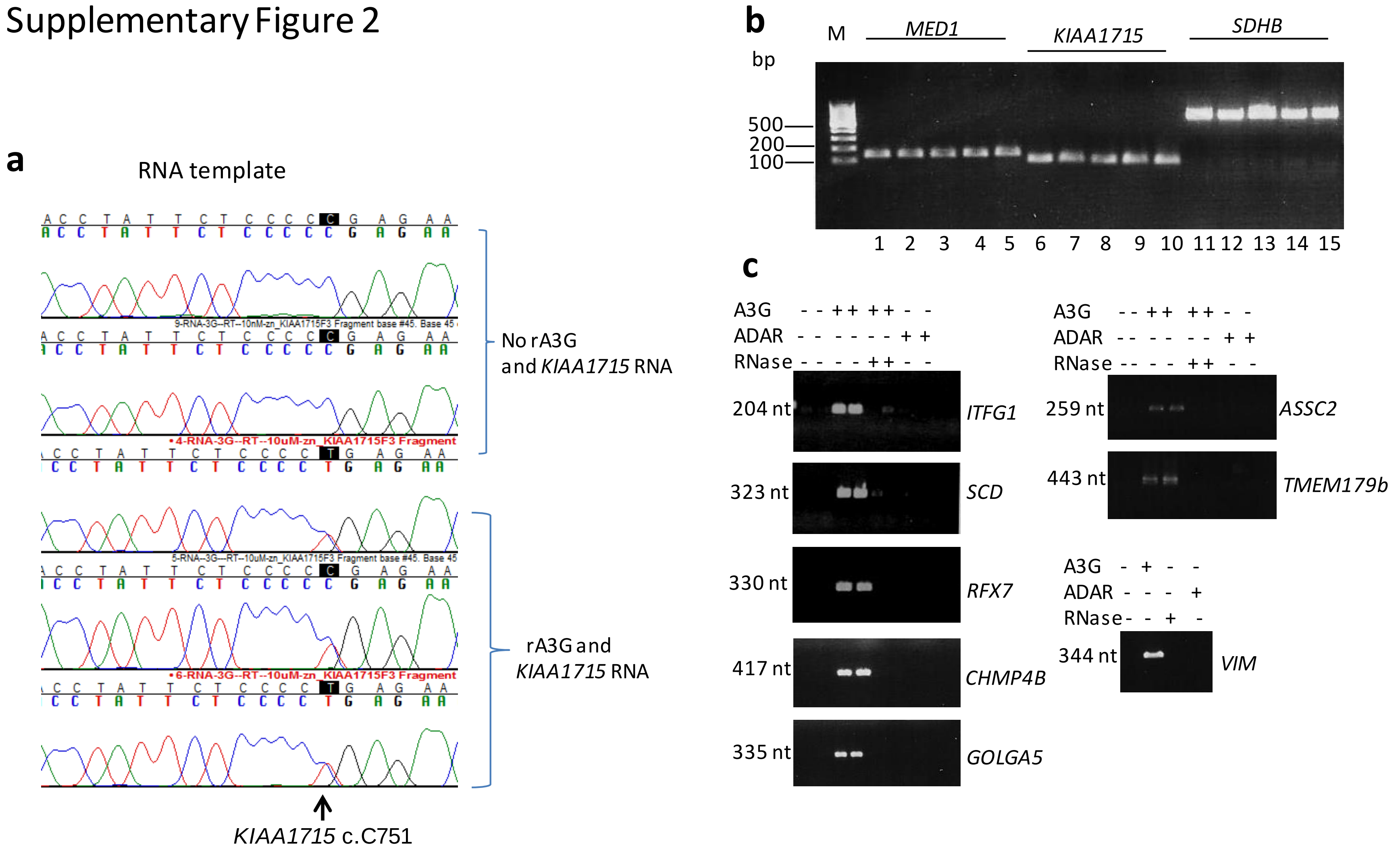

Supplementary Fig. 2. Recombinant APOBEC3G co-purifies with bound RNAs . (a) Chromatograms of cDNA (in duplicate) RT-PCR products of *KIAA1715* RNA from in vitro editing assay in the absence or presence of purified A3G confirms site-specific C>U RNA editing (edited C is shaded black) in selected genes. **(b)** Gel electrophoresis showing RT-PCR products of *MED1, KIAA1715* and *SDHB* RNAs that were amplified from in vitro RNA editing reactions containing rA3G only (lanes 1-3, 6-8 and 11-13). Lanes 4,5,9,10 and 14,15 are controls from 293T cells transiently expressing EV or A3G. **(c)** Gel electrophoresis showing RT-PCR products of *ITFG1, SCD, RFX7, CHMP4B, GOLGA5, ASSC2, TMEM179b* and *VIM* RNAs that were amplified from in vitro RNA editing reactions containing rA3G with or without RNase or ADAR enzyme (n=2 except *VIM,* n=1*).*

Supplementary Figure 3

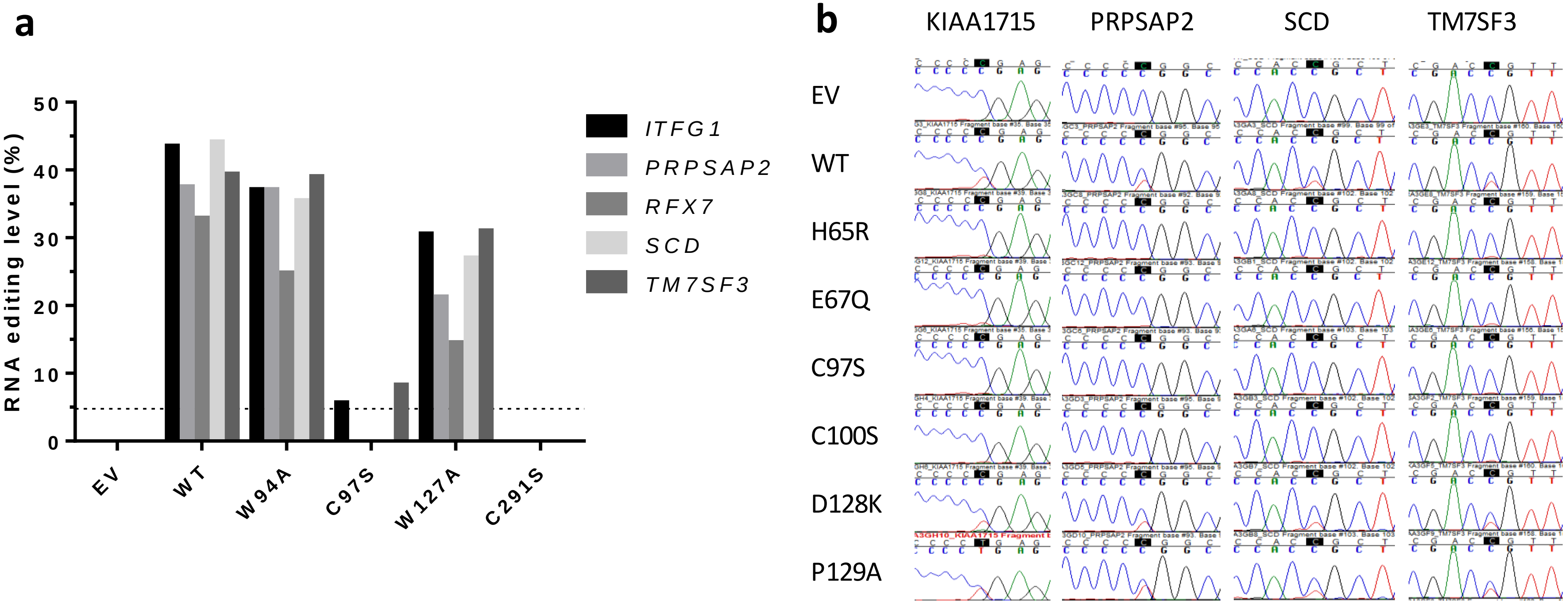

Supplementary Fig. 3. Site-directed mutagenesis of APOBEC3G shows requirement of both N- and C–terminal domain zinc coordinating site residues for site-specific RNA deamination. (**a**) Site-directed mutagenesis of APOBEC3G shows requirement of both N- and C–terminal domain Zinc coordinating residues for site-specific RNA editing. Bars represent percentage of T peaks in Sanger sequencing traces shown in Fig. 4a (n=1). (**b**) Representative chromatogram of cDNA of PCR products from control/WT/mutant A3G transfectants shown in Fig. 4d demonstrating the effect of A3G-NTD and CTD mutations on C>U RNA editing (edited C is shaded black) of *KIAA1715, PRPSAP2, SCD and TM7SF3.*

Supplementary Figure 4

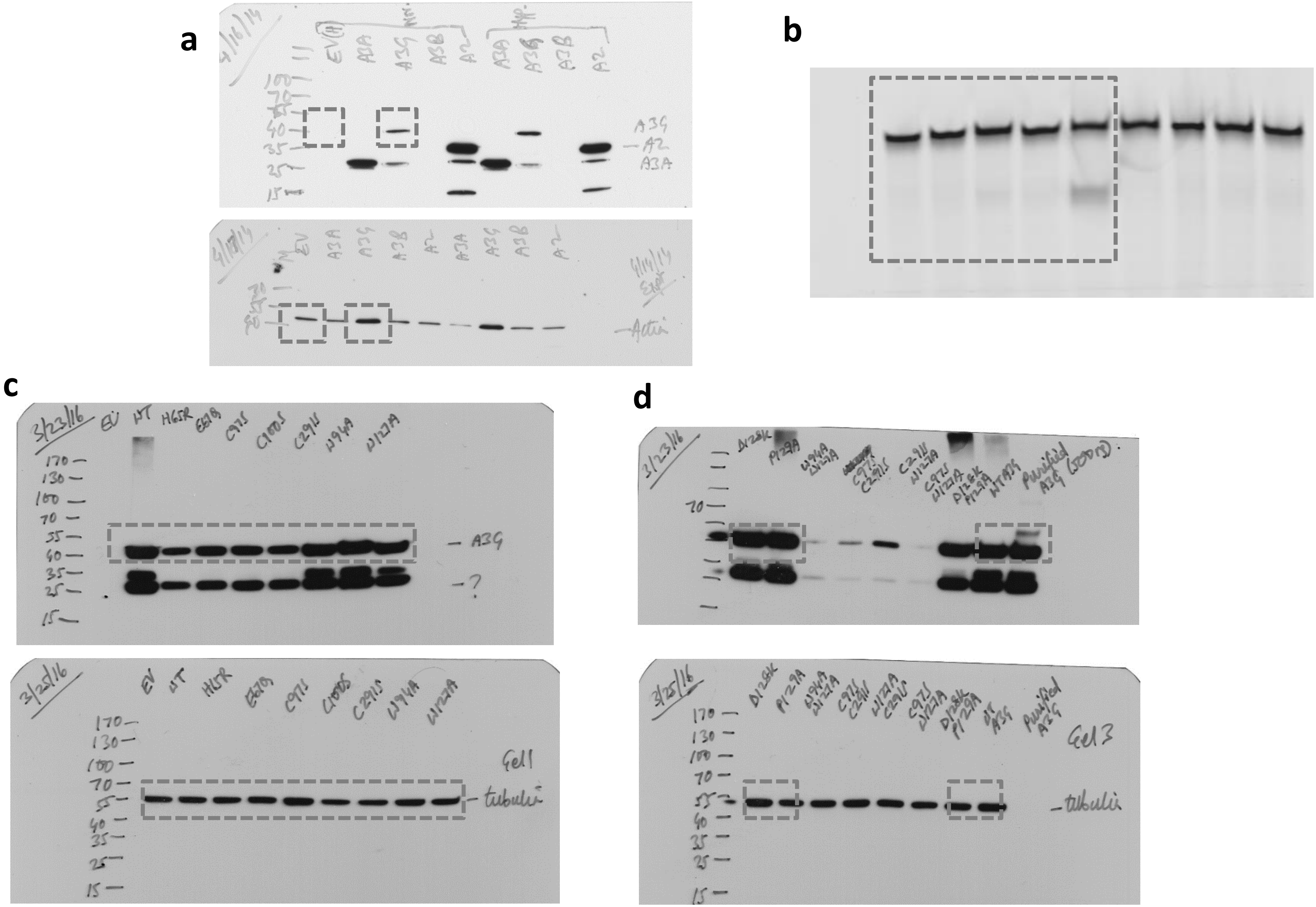

Supplementary Fig. 4. Full-length versions of immunoblots and gels. (a) Uncropped immunoblot shown in Supplementary Fig. S1. **(b)** Uncropped version of the gel depicting the DNA deamination assay in Fig. 3b. **(c)** Uncropped immunoblot shown in Fig. 4c. **(d)** Uncropped immunoblot shown in Fig. 3a (dotted box on the right). Dotted gray boxes depict the cropped regions shown in the main/supplementary figures
